# Influence of Dietary Nutrient Intake on Episodic Memory Across the Adult Life Span

**DOI:** 10.3389/fnagi.2021.724595

**Published:** 2021-08-30

**Authors:** Selene Cansino, Frine Torres-Trejo, Cinthya Estrada-Manilla, Adriana Flores-Mendoza, Gerardo Ramírez-Pérez, Silvia Ruiz-Velasco

**Affiliations:** ^1^Laboratory of NeuroCognition, Faculty of Psychology, National Autonomous University of Mexico, Mexico City, Mexico; ^2^Applied Mathematics and Systems Research Institute, National Autonomous University of Mexico, Mexico City, Mexico

**Keywords:** episodic memory, source memory, recollection, nutrients, moderator analysis

## Abstract

The aim of the study was to identify nutrients that have the ability to impact brain functioning and, as a consequence, influence episodic memory. In particular, we examined recollection, the ability to recall details of previous experiences, which is the episodic memory process most affected as age advances. A sample of 1,550 healthy participants between 21 and 80 years old participated in the study. Nutritional intake was examined through a food frequency questionnaire and software developed to determine the daily consumption of 64 nutrients based on food intake during the last year. Recollection was measured through a computerized source memory paradigm. First, we identified which nutrients influence recollection across the entire adult life span. Then, moderator analyses were conducted by dividing the sample into young (21–40 years old), middle-aged (41–60 years old) and older (61–80 years old) adults to establish in which life stage nutrients influence episodic memory. Across the adult life span, recollection accuracy was shown to benefit from the intake of sodium, heme, vitamin E, niacin, vitamin B6, cholesterol, alcohol, fat, protein, and palmitic, stearic, palmitoleic, oleic, gadoleic, alpha-linoleic and linoleic acid. The effects of energy, maltose, lactose, calcium and several saturated fatty acids on recollection were modulated by age; in older adults, the consumption of these nutrients negatively influenced episodic memory performance, and in middle-aged adults, only lactose had negative effects. Several brain mechanisms that support episodic memory were influenced by specific nutrients, demonstrating the ability of food to enhance or deteriorate episodic memory.

## Introduction

Memory is often considered the ability to remember one’s own past. This kind of memory is known as episodic memory, a term introduced by Tulving (1972) to differentiate this type of memory from semantic memory. The main characteristic that distinguishes episodic memory from semantic memory is that the latter stores general knowledge lacking details about how the information was learned. Conversely, episodic memory stores specific personal events along with the contextual details that accompanied the event. However, within the realm of episodic memory, it is possible to remember events that do not include contextual details by means of familiarity, a process that only provides a vague sense that an event has previously occurred (Mandler, 1980). This process is distinguished from recollection by the ability to remember what happened, where and when (Tulving, 2002). Therefore, only recollection may be conceived as truly episodic.

Consequently, assessing whether familiarity or recollection processes are taking place is essential for the characterization of the patterns of episodic memory functioning. This is particularly important when episodic memory is assessed across the adult life span because it has been demonstrated that recollection but not familiarity decreases drastically with advancing age (Spencer and Raz, 1995; Cansino et al., 2013). Recollection deficits are manifested in everyday living by situations such as forgetting if one has done something or not, remembering situations in erroneous contexts or being unable to remember how to perform a specific task. As these deficits increase, even simple duties become more difficult, and individuals gradually start to lose control of their own life.

For that reason, the identification of factors that may positively or negatively influence the course of episodic memory decline across the adult life span is an important field of study. Nutrient intake is a potentially related factor because of its direct influence on brain function (Gómez-Pinilla, 2008). The search for nutrients that may influence cognitive function has followed several routes. One of them is through intervention studies; in humans, this kind of study has been conducted mainly on individuals diagnosed with mild cognitive impairment or Alzheimer’s disease (for a review see McGrattan et al., 2020). Research with healthy older adults has not provided consistent findings. A meta-analysis that included 24 trials found no significant effects of omega-3 fatty acids, vitamin B or vitamin E on general cognition (Forbes et al., 2015), whereas another meta-analysis study (McEvoy et al., 2019) that included 15 trials showed that dietary modification improved global cognition but not memory. However, two trials found that polyphenols from blueberries in older adults (Whyte et al., 2018) and cocoa in young adults (Lamport et al., 2020) benefit episodic memory, measured with lists of words. Although intervention studies are useful to investigate the potential benefits of specific nutrients on cognition, global conclusions from several studies are difficult to draw due to methodological differences. Moreover, most of these studies rely on measurements of general cognitive function that may not be sufficiently sensitive to nutrient interventions because cognition does not decline as a whole but in specific domains (Glisky, 2007). Additionally, nutrient intervention through the use of supplements, often used in intervention studies, may be less effective than nutrients obtained from food (e.g., Morris et al., 2002).

Another approach to studying nutrient effects on cognition is through the conduction of observational studies. These studies are useful to detect effects that might not be possible to identify otherwise. Furthermore, obtaining negative results in observational studies prevents investigators from spending time and effort conducting randomized studies on nutrients that failed to provide any benefit (Cooper, 2014). Observational studies have mostly investigated whether specific dietary patterns may prevent cognitive decline. One meta-analysis (Mottaghi et al., 2018) that included six studies found that increased fruit and vegetable intake was associated with a lower risk of cognitive impairment. A systematic review (van de Rest et al., 2015) found that among several dietary patterns, adherence to a Mediterranean diet was associated with reduced cognitive decline and dementia. Likewise, Loughrey et al. (2017) found in a meta-analysis of 15 studies conducted in healthy older adults that a Mediterranean diet was positively associated with episodic memory and general cognition. Although it is useful to determine that dietary patterns, such as the Mediterranean diet, have favorable effects on cognition, a dietary pattern only provides general information about the benefits of consuming a mix of foods. However, it is impossible to discern which elements of each food are providing these benefits. The absence of this information impedes the identification of the mechanisms involved in the brain benefits that manifest in the form of cognitive functioning. Moreover, a dietary pattern such as the Mediterranean diet only represents a general guide for choosing healthy food because it varies considerably across and within populations.

Therefore, examining the direct influence of specific nutrients on cognition is a step forward in identifying the nutrients that actually have an effect on brain functioning and in elucidating the mechanisms that may underlie these effects. The purpose of the present study was twofold: first, to identify the potential influence of 64 dietary intake nutrients on episodic memory, particularly recollection, in an adult life span sample; second, to assess whether the influence of any of these nutrients on recollection is moderated by individuals’ age. Several physiological changes take place with advancing age that may modify the effects of nutrients on brain function. For example, metabolic rates, which are responsible for converting food into energy, decrease due to body composition alterations, such as the loss of muscle mass and organ weight (Henry, 2000). To test age as a moderator variable, the entire sample was divided into young (21–40 years old), middle-aged (41–60 years old) and older (61–80 years old) adults because there is evidence that nutrition requirements considerably change in these critical adult life stages (e.g., Ilich and Brownbill, 2010).

Previous observational studies have been conducted mainly in older adults, and there are very few that have been conducted in adults over 50 years old (van de Rest et al., 2015). Thus, one novelty of the current study is that we analyzed the effects of nutrients in a life span sample. Moreover, by dividing the sample into young, middle-aged and older adults, we were able to distinguish, at each of these life stages, how age moderated the effects of nutrients on episodic memory. This examination has not been performed before. Another uniqueness of the current study is that we measured episodic memory with a computerized task; previous observational studies have employed paper-and-pencil procedures to measure general cognition or episodic memory. Notably, paper-and-pencil methods are less accurate and controllable. Furthermore, we employed a source memory paradigm that reliably measured recollection, the main process within episodic memory that deteriorates with advancing age. Another novelty is that we simultaneously assessed the effects of numerous nutrients on episodic memory in the same sample, an approach that allowed us to contrast the influence of each nutrient on the same conditions.

Based on previous findings (for reviews see Gómez-Pinilla, 2008; Spencer et al., 2017), we expected that several nutrients that have been identified as positively or negatively affecting brain function, such as calcium, zinc, selenium, copper, iron, vitamin B, vitamin D, vitamin E, saturated fatty acids and omega three fatty acids, would influence recollection processes. Additionally, we expected that age, as revealed by previous studies, would moderate the effects of some nutrients on recollection, such as the effects of calcium, vitamin D (Rossom et al., 2012) and saturated fatty acids (Greenwood and Winocur, 2005).

## Materials and Methods

### Participants

A sample of 1,550 healthy adults between 21 and 80 years old participated in this study (780 women, 770 men). The entire sample was divided into three groups according to participants’ age into young (21–40 years old), middle-aged (41–60 years old) and older (61–80 years old) adults. Participant characteristics and scores on neuropsychological screening tests are displayed for the three age groups in Table 1. Subjects were recruited through advertisements, flyers, appeals to community groups and word of mouth. The inclusion criteria were a minimum of eight years of education, normal or corrected-to-normal vision, a score ≤ 20 on the Beck Depression Inventory (BDI) (Beck, 1987), a score ≥ 24 on the Mini-Mental State Exam (MMSE) (Folstein et al., 1975), and a score ≥ 26 on the vocabulary subtest of the Wechsler Adult Intelligence Scale-Revised (WAIS-R) (Wechsler, 1981). These performance scores ensure that participants were not afflicted by depression, dementia, or intellectual difficulties. The exclusion criteria were addiction to drugs or alcohol, use of medication that acts on the nervous system in the previous 6 months, neurological or psychiatric diseases, or head trauma. All participants provided informed consent and received a monetary reward for their participation. The study was approved by the Bioethics Committee of the School of Medicine at the National Autonomous University of Mexico. All experiments were performed in accordance with the Declaration of Helsinki. A power analysis was conducted with the software G^∗^Power version 3.1.9.2 to estimate the sample size required to conduct linear multiple regression analysis with two predictors and one interaction term. The parameters used to calculate the sample size were an expected effect size *f*^2^ = 0.35, an alpha level of.05, a power value of.95 and three predictors. The results suggested a total sample size of 54 participants.

**TABLE 1:** Participant characteristics and scores on neuropsychological tests in the three age groups.

	Young (*n* = 521) M (*SD*)	Middle-aged (*n* = 512) M (*SD*)	Older (*n* = 517) M (*SD*)
Age (years)	29.5 (6.3)	50.8 (5.3)	70.2 (5.4)
Sex (women/men)	258/263	257/255	257/255
Education (years)	15.9 (2.8)	14.8 (4.6)	13.3 (4.5)
Vocabulary (WAIS-R)	12.8 (1.6)	12.7 (1.7)	12.8 (1.8)
Mini Mental Stare Examination	29.0 (1.1)	28.6 (1.3)	28.2 (1.5)
Beck Depression Inventory	6.1 (5.0)	6.4 (5.0)	7.2 (5.1)
Body mass index (k/m^2^)	25.1 (3.9)	27.6 (4.3)	26.7 (3.8)
Glucose (mg/dL)	91.0 (30.4)	101.0 (37.6)	108.0 (41.3)
Cholesterol (mg/dL)	168.6 (28.2)	186.9 (34.7)	191.7 (36.4)
Triglycerides (mg/dL)	186.7 (123.5)	238.1 (130.9)	238.3 (131.3)
Heart rate (bpm)	70.0 (11.5)	70.2 (10.4)	68.6 (11.7)
Systolic blood pressure (mmHg)	107.9 (12.7)	118.3 (16.4)	127.5 (17.9)
Diastolic blood pressure (mmHg)	69.7 (9.3)	76.4 (10.1)	75.4 (10.5)
Mean arterial pressure (mmHg)	82.4 (9.6)	90.3 (11.6)	92.8 (11.8)

*WAIS-R, Wechsler Adult Intelligence Scale-Revised.*

### Instruments

Nutrient intake was measured with the Food Frequency Questionnaire (FFQ) (Hernández-Ávila et al., 1998), a semiquantitative instrument composed of 116 food items designed to assess dietary intake. The frequency of consumption of each food item over the previous year was measured using 10 frequency categories ranging from never, less than once a month, 1–3 times per month, once a week, 2–4 times per week, 5–6 times per week, once a day, 2–3 times per day, 4–5 times per day and six times per day. The FFQ was developed in many steps following the method proposed by Willett et al. (1985). First, food items were identified from a random sample of families in Mexico City (Hernández et al., 1983) through 24-h recalls and home visits where food items consumed were weighed and measured. Then, dietitians and nutritionists identified possible food items that could be missing; the FFQ was then created and piloted with a similar sample. The nutrient content of each food item was estimated per average unit (serving size) by the United States Department of Agriculture (USDA) and complemented by a National Institute of Nutrition database (Chávez et al., 1996). The reproducibility of the FFQ was assessed for a period of 1 year, and its validity was estimated by comparing results from the FFQ with those obtained from four 4-day 24-h recalls during a period of 1 year (Hernández-Ávila et al., 1998) and with serum blood levels of some of the nutrients (Romieu et al., 1999). Nutrient intake was estimated using the software Evaluation System of Nutritional Habits and Nutrient Consumption (SNUT) developed by the National Institute of Public Health (Hernández-Ávila et al., 2000). The validity and reproducibility of the FFQ have been confirmed for individuals living in Mexico City (e.g., Hernández-Ávila et al., 1998).

### Stimuli

A total of 122 color images of common objects were employed in the source memory task: 50% represented natural objects, and the remaining images were of artificial objects. Twelve images were employed in a practice session, and two were presented at the beginning of the encoding and retrieval phases from the source memory paradigm; data from these 14 images were not analyzed. From the remaining 108 images, 72 were randomly selected (equal proportion of natural and artificial objects) for each participant to be presented during the encoding phase, and the entire set of 108 images was randomly presented at the retrieval phase. The images subtended vertical and horizontal visual angles ranging from 2.9° to 4.3° and were displayed on a white background.

### Procedure

Before being invited to attend the first session, potential participants were asked prescreening questions to inquire whether they fulfilled the inclusion criteria. During the first session, participants were interviewed to further determine if they satisfied the inclusion criteria. Then, participants performed the WAIS-R Vocabulary subtest, the MMSE and the BDI, and their vision was tested. Participants who were eligible for the study were requested to provide their informed consent. Afterward, participants were further interviewed about their health and lifestyle, followed by the application of the FFQ. Finally, participants’ weight and height were measured. The first session took place in a silent room in which only the participant and the experimenter were present. One week after the first session, participants attended the second session. First, participants’ glucose, cholesterol and triglycerides were measured in a non-fasting state with the Accutrend Plus System (Roche Diagnostics, Rotkreuz, Switzerland). These measures were conducted in a counterbalanced order. Then, blood pressure and heart rate were measured with a digital upper arm sphygmomanometer (Hem-712C, Omron, Kyoto, Japan). Mean arterial pressure (MAP) was estimated as [(2 × diastolic blood pressure) + systolic blood pressure]/3. Afterward, participants performed a working memory task (data not shown) and a source memory task in a sound-dampened chamber while seated in a high-back armchair located 100 cm away from the monitor screen. A response panel was located on a platform placed on either the left or right arm of the chair at a comfortable distance, according to the participants’ handedness. Stimulus presentation and response recording were controlled by E-Prime software v1.0 (Psychological Software Tools, Pittsburgh, PA, United States).

#### Source Memory Paradigm

This paradigm, introduced by Cansino et al. (2002), allows for the objective measurement of recollection processes because participants are requested to report the exact spatial context in which each item was previously presented during a study phase. The task consisted of an encoding phase and a retrieval phase. During the encoding phase, a cross divided the screen into quadrants; the center of the cross was in the middle of the screen. Images were randomly displayed in one of the quadrants with the same probability of appearing in each of them. The images were displayed at a distance ranging from 0.5° to 1.25° away from the axes of the cross dividing the screen. Each trial started with the presentation of an image for 1,000 ms followed by a 3,000 ms period when only the cross remained on the screen. The task consisted of classifying whether the images represented a natural or an artificial object by pressing one of two keys. Participants were able to respond during a period of 3,500 ms following the onset of the stimulus. The response panel consisted of four keys arranged in two columns of two rows, each to be pressed by the index and middle finger, and a fifth key located in the lower portion of the response panel to be pressed by the thumb. The four keys represented the quadrants of the screen where the images were presented during the encoding phase. The two keys in the second row were used during the encoding phase, while all five keys were used during the retrieval phase. During the retrieval phase, the screen was not divided into quadrants, and the images were presented in the center of the screen. The same timing used in the encoding task was used in the retrieval task. Participants were asked to judge whether the image was new or old. If the image was old, they indicated the quadrant where the image was originally presented during the encoding phase by pressing one of the four keys that represented each quadrant of the screen. If the image was new, participants used their thumb to press the lower (fifth) key on the response panel. Participants were instructed to randomly select one of the four quadrants if they were confident that the image was old but were unable to remember its exact position. The participants performed a training session that involved a shorter version of the encoding and retrieval tasks to learn how to use the response panel.

### Data Analysis

Source memory accuracy or recollection accuracy was estimated as the percent of recognition hits accompanied by a correct source response. A set of 64 nutrients was initially considered a potential predictor of recollection accuracy. All variables were examined with descriptive analyses, and those with skewness exceeding + 2 were natural log-transformed. To determine which nutrients significantly predict recollection performance, we conducted linear regression analyses in the whole sample between each nutrient and recollection accuracy. To control for multiple regressions, the Benjamini-Hochberg procedure was applied with a false discovery rate of.05 (Benjamini and Hochberg, 1995). Non-corrected probabilities are reported. This approach was chosen because the purpose of our study was to analyze the possible influence of each nutrient on episodic memory. Multiple regression analysis including all nutrients in the model is not a suitable procedure due to nutrient multicollinearity, which will interfere with determining the precise effect of each predictor and may provide unreliable coefficient estimates for individual predictors. Developing data with reduction techniques was not appropriate because we were interested in the effects of independent nutrients that can be directly compared with nutrients examined in other studies.

To estimate whether age, defined as three age groups (young, middle-aged and older adults), is a moderator of the influence of each nutrient on episodic memory, we conducted moderator analysis between each nutrient and recollection accuracy. The multiple regression models included each nutrient, age group and the interaction term nutrient × age group. Nutrient variables were centered for these analyses. If the interaction term was significant in the regression model, we estimated whether the slope for the nutrient was significantly different between middle-aged and older adults since this difference was not estimated by the model. Additionally, bootstrapped tests with 20,000 replications were performed to estimate whether the B with 95% confidence intervals remained significant. The nutrients that influence recollection accuracy that were significantly moderated by age group were analyzed through one-way analysis of variance (ANOVA) to examine whether nutrient consumption differs across age groups. Significant differences among age groups were further analyzed by Tukey’s honest significance difference (HSD) tests. All analyses were conducted using Stata v. 16 (Texas, United States).

## Results

**FIGURE 1:**
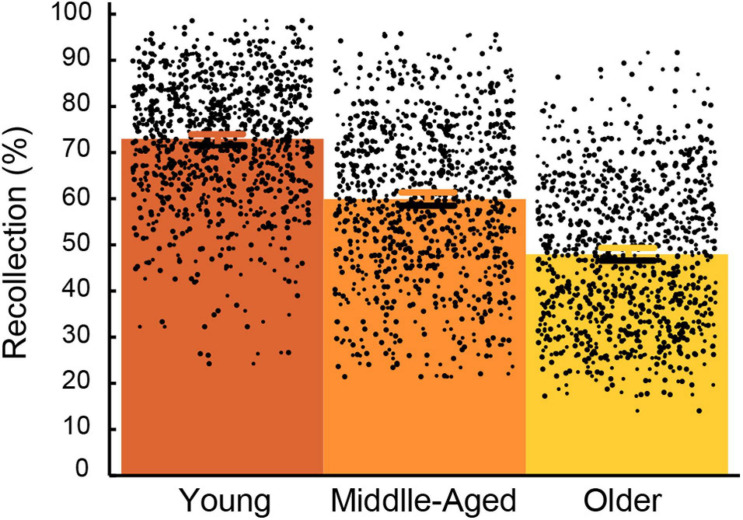
Mean and individual recollection performance in young (21–40 years old), middle-aged (41–60 years old) and older adults (61–80 years old). Error bars represent the 95% confidence intervals for the mean.

Recollection accuracy in each age group is displayed in Figure 1. The descriptive analyses conducted on all variables are depicted in Supplementary Material Supplementary Table 1. After being log-transformed, all variables had a skewness below + 2, except for eicosatetranoic acid ω-3 (skewness = 2.06) and eicosapentaenoic acid ω-3 (skewness = 2.42). The results of the linear regression analyses conducted for each nutrient and recollection accuracy are shown according to the group of nutrients in Table 2 (general nutrients), Table 3 (carbohydrates), Table 4 (micronutrients), Table 5 (carotenoids), and Table 6 (fatty acids). The results revealed that 26 nutrients significantly predicted recollection accuracy; however, after applying the Benjamini-Hochberg procedure, thiamine, vitamin B12, eicosatetranoic acid and phosphorus were no longer significant predictors.

**TABLE 2:** Linear regressions between each nutrient and recollection accuracy (%).

	*b*	*SE b*	β	*t*	*P*
**Total macronutrients**					
Carbohydrates (g)	0.006	0.004	0.033	1.332	0.183
Protein (g)	0.047	0.015	0.076	3.125	0.002*
Fat (g)	0.074	0.011	0.152	6.499	0.000*
**Other consumptions**					
Energy (kcal)	0.002	0.001	0.090	3.682	0.000*
Cholesterol (g)^a^	2.306	0.810	0.070	2.848	0.004*
Alcohol (g)^a^	2.801	0.622	0.114	4.501	0.000*
Caffeine (g)	0.002	0.003	0.014	0.570	0.569
Nitrate (g)^a^	–0.209	0.819	–0.006	–0.255	0.799

^a^Log-transformed variable.

*Significant p-values after the Benjamini-Hochberg method for controlling the false discovery rate.

**TABLE 3:** Linear regressions between each type of carbohydrate and recollection accuracy (%).

			*b*	*SE b*	β	*t*	*P*
**Simple Carbohydrates**							
	Monosaccharides						
		Glucose (g)^a^	0.609	0.942	0.017	0.647	0.518
		Fructose (g)	0.005	0.028	0.004	0.166	0.868
	Disaccharides						
		Maltose (g)	0.337	0.518	0.017	0.652	0.515
		Sucrose (g)	0.024	0.022	0.029	1.078	0.281
		Lactose (g)	0.017	0.042	0.010	0.400	0.689
**Complex carbohydrates**							
		Starch (g)	0.004	0.011	0.010	0.392	0.695
		Soluble fiber (g)	0.039	0.112	0.009	0.350	0.726
		Insoluble fiber (g)	–0.007	0.062	–0.003	–0.113	0.910

*^a^Log-transformed variable.*

**TABLE 4:** Linear regressions between each micronutrient and recollection accuracy (%).

	*b*	*SE b*	β	*t*	*P*
**Macrominerals**					
Calcium (mg)	0.000	0.001	0.004	0.177	0.859
Phosphorus (mg)	0.002	0.001	0.051	2.100	0.036
Magnesium (mg)	–0.001	0.004	–0.006	–0.250	0.803
Potassium (mg)	–0.000	0.000	–0.005	–0.171	0.864
Sodium (mg)	0.002	0.001	0.073	3.034	0.002*
**Trace minerals**					
Copper (mg)^a^	–1.026	1.150	–0.023	–0.892	0.372
Iron (mg)	0.102	0.091	0.028	1.119	0.263
Heme (g)^a^	7.480	1.868	0.099	4.005	0.000*
Manganese (mg)^a^	–0.457	0.450	–0.021	–1.015	0.310
Selenium (mcg)	0.035	0.018	0.044	1.880	0.060
Zinc (mg)^a^	0.459	0.999	0.012	0.459	0.646
**Fat-soluble vitamins**					
Retinol (UI)^a^	1.295	0.707	0.046	1.833	0.067
Vitamin D (UI)	–0.005	0.003	–0.033	–1.614	0.107
Alpha-tocopherol (mg)^a^	4.516	1.136	0.098	3.976	0.000*
Beta-tocopherol (mg)	3.342	1.153	0.072	2.897	0.004*
Gamma-tocopherol (mg)	0.360	0.047	0.176	7.713	0.000*
Delta-tocopherol (mg)^a^	7.999	1.077	0.181	7.425	0.000*
Vitamin K (mcg)^a^	1.593	0.844	0.048	1.886	0.059
**Water-soluble vitamins**					
Thiamine (mg)	1.546	0.658	0.059	2.350	0.019
Riboflavin (mg)	0.980	0.581	0.042	1.686	0.092
Niacin (mg)	0.178	0.058	0.075	3.048	0.002*
Pantothenic acid (mg)^a^	–1.188	0.821	–0.037	–1.446	0.148
Vitamin B6 (mg)^a^	1.973	0.596	0.082	3.313	0.001*
Folate (mcg)^a^	–1.151	0.645	–0.046	–1.785	0.074
Vitamin B12 (mcg)^a^	1.848	0.828	0.056	2.231	0.026
Vitamin C (mg)	–0.002	0.003	–0.018	–0.857	0.392

^a^Log-transformed variable.

*Significant p-values after the Benjamini-Hochberg method for controlling the false discovery rate.

**TABLE 5:** Linear regressions between each carotenoid type and recollection accuracy (%).

	*b*	*SE b*	B	*t*	*P*
**Carotene**					
Carotenes (UI)^a^	–0.226	0.573	–0.008	–0.395	0.693
Alpha-carotene (mcg)^a^	–0.760	0.431	–0.045	–1.762	0.078
Beta-carotene (mcg)	–0.000	0.000	–0.031	–1.171	0.242
Lycopene (mcg)^a^	0.944	0.635	0.039	1.487	0.137
**Xanthophyll**					
Beta-cryptoxanthin (mcg)	–0.001	0.001	–0.028	–1.359	0.174
Lutein & zeaxanthine (mcg)^a^	–0.981	0.638	–0.040	–1.536	0.125

*^a^Log transformed variable.*

**TABLE 6:** Linear regressions between each fatty acid and recollection accuracy (%).

			*b*	*SE b*	β	*t*	*P*
**Saturated**							
	Butyric acid (g)		1.381	1.760	0.020	0.785	0.433
	Caproic acid (g)		0.569	2.765	0.005	0.206	0.837
	Caprylic acid (g)		2.777	5.006	0.014	0.555	0.579
	Capric acid (g)		3.619	2.132	0.042	1.697	0.090
	Lauric acid (g)		4.461	1.732	0.063	2.576	0.010*
	Myristic acid (g)		1.479	0.471	0.077	3.143	0.002*
	Palmitic acid (g)		0.472	0.077	0.147	6.143	0.000*
	Stearic acid (g)		1.106	0.170	0.157	6.505	0.000*
**Unsaturated**							
	Monounsaturated						
		Palmitoleic acid ω-7 (g)	3.140	0.608	0.125	5.167	0.000*
		Oleic acid ω-9 (g)	0.255	0.045	0.129	5.601	0.000*
		Gadoleic acid ω-11 (g)	15.424	4.870	0.076	3.167	0.002*
	Polyunsaturated						
		Alpha-linoleic acid ω-3 (g)^a^	6.951	1.660	0.108	4.186	0.000*
		Eicosatetraenoic acid ω-3 (g)^a^	19.947	9.010	0.051	2.214	0.027
		Eicosapentaenoic acid ω-3 (g)^a^	–7.543	6.882	–0.026	–1.096	0.273
		Docosahexaenoic acid ω-3 (g)	–0.791	2.853	–0.007	–0.277	0.782
		Linoleic acid ω-6 (g)	0.683	0.078	0.189	8.794	0.000*

^a^Log-transformed variable.

*Significant p-values after the Benjamini-Hochberg method for controlling the false discovery rate.

**FIGURE 2:**
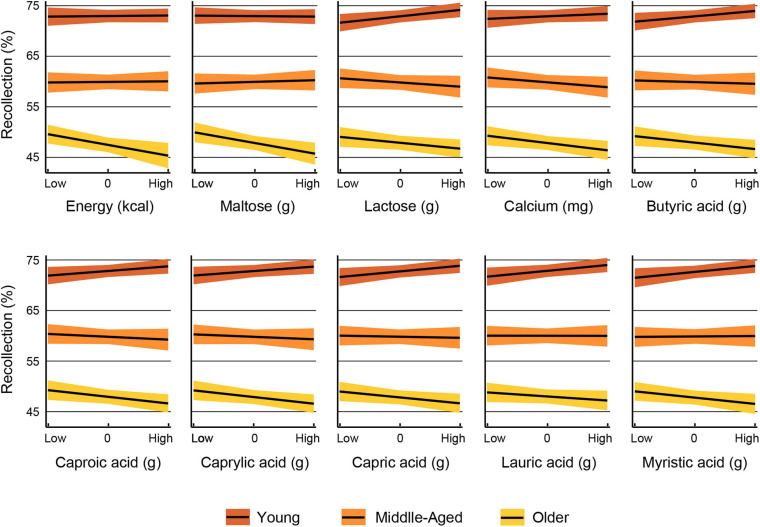
The interaction is displayed for nutrients for which recollection performance was significantly moderated by age group. The relationship between each nutrient and recollection accuracy by age group is shown for nutrients at levels one standard deviation below the mean (low), at the mean (centered to zero) and one standard deviation above the mean (high). The color bands represent the 95% confidence interval.

The results of the multiple regression analyses in which the interaction term was significant are displayed in Tables 7, 8. These analyses revealed that the influence of 10 nutrients on recollection accuracy was significantly moderated by age group. The interactions are depicted in Figure 2. In all nutrient models, a significant slope difference was observed between young and older adults. Additionally, the slope for lactose significantly differed between young and middle-aged adults (Table 7). Further analyses were conducted to determine whether the slopes for each nutrient differ between middle-aged and older adults. The results revealed that only the slopes for energy [*F*(1,1544) = 4.07, *p* = 0.044] and maltose [*F*(1,1544) = 5.27, *p* = 0.022] differed significantly between these two age groups.

**TABLE 7:** Moderator analyses results.

			B	β	*SE* B	*P*	95% CI for B
Energy (kcal)			–0.000	0.006	0.001	0.831	−0.001 0.001
		Middle-aged	–13.026	–0.331	0.962	0.000	−14.913 −11.138
		Older	–25.487	–0.648	0.963	0.000	−27.376 −23.598
		Middle-aged × energy	0.000	0.000	0.001	0.999	−0.002 0.002
		Older × energy	–0.003	–0.060	0.001	0.026	−0.005 −0.000
		Intercept	72.949		0.625	0.000	71.723 74.175
Maltose (g)			–0.107	–0.005	0.547	0.845	−1.180 0.966
		Middle-aged	–13.065	–0.332	0.950	0.000	−14.929 −11.201
		Older	–25.221	–0.642	0.927	0.000	−27.040 −23.402
		Middle-aged × maltose	0.454	0.013	0.937	0.628	−1.384 2.293
		Older × maltose	–2.134	–0.056	0.996	0.032	−4.089 −0.180
		Intercept	72.988		0.606	0.000	71.799 74.177
Lactose (g)			0.114	0.068	0.048	0.016	0.210 0.207
		Middle-aged	–13.015	–0.330	0.954	0.000	−14.886 −11.145
		Older	–24.882	–0.633	0.928	0.000	−26.703 −23.062
		Middle-aged × lactose	–0.190	–0.063	0.082	0.021	−0.350 −0.029
		Older × lactose	–0.220	–0.078	0.075	0.004	−0.367 −0.072
		Intercept	72.847		0.608	0.000	71.654 74.040
Calcium (mg)			0.001	0.027	0.002	0.378	−0.002 0.004
		Middle-aged	–13.050	–0.331	0.952	0.000	−14.917 −11.183
		Older	–25.048	–0.637	0.927	0.000	−26.867 −23.230
		Middle-aged × calcium	–0.004	–0.044	0.002	0.109	−0.009 0.001
		Older × calcium	–0.005	–0.062	0.002	0.025	−0.010 −0.001
		Intercept	72.923		0.609	0.000	71.728 74.118
Butyric acid (g)			4.000	0.057	1.995	0.045	0.087 7.913
		Middle-aged	–12.964	–0.329	0.957	0.000	−14.842 −11.086
		Older	–24.890	–0.633	0.930	0.000	−26.714 −23.067
		Middle-aged × butyric acid	–5.254	–0.042	3.493	0.133	−12.106 1.598
		Older × butyric acid	–8.843	–0.075	3.168	0.005	−15.056 −2.630
		Intercept	72.844		0.611	0.000	71.645 74.044

**TABLE 8:** Moderator analyses results.

			B	β	*SE* B	*P*	95% CI for B
Caproic acid (g)			5.533	0.050	3.202	0.084	−0.748 11.814
		Middle-aged	–13.023	–0.331	0.955	0.000	−14.896 −11.151
		Older	–24.903	–0.634	0.928	0.000	−26.724 −23.082
		Middle-aged × caproic acid	–8.918	–0.045	5.491	0.105	−19.689 1.853
		Older × caproic acid	–13.440	–0.073	5.019	0.007	−23.284 −3.595
		Intercept	72.876		0.609	0.000	71.682 74.070
Caprylic acid (g)			9.578	0.048	5.717	0.094	−1.637 20.792
		Middle-aged	–13.003	–0.330	0.956	0.000	−14.879 −11.127
		Older	–24.914	–0.634	0.929	0.000	−26.737 −23.091
		Middle-aged × caprylic acid	–14.846	–0.041	9.874	0.133	−34.215 4.523
		Older × caprylic acid	–23.870	–0.071	9.101	0.009	−41.721 −6.018
		Intercept	72.865		0.611	0.000	71.666 74.063
Capric acid (g)			5.151	0.060	2.480	0.038	0.286 10.016
		Middle-aged	–12.901	–0.327	0.958	0.000	−14.781 −11.022
		Older	–24.885	–0.633	0.932	0.000	−26.714 −23.057
		Middle-aged × capric acid	–6.140	–0.040	4.199	0.144	−14.377 2.097
		Older × capric acid	–10.561	–0.073	3.952	0.008	−18.312 −2.810
		Intercept	72.796		0.615	0.000	71.589 74.003
Lauric acid (g)			4.365	0.062	2.048	0.033	0.348 8.382
		Middle-aged	–12.845	–0.326	0.960	0.000	−14.728 −10.961
		Older	–24.846	–0.632	0.935	0.000	−26.680 −23.013
		Middle-aged × lauric acid	–4.439	–0.035	3.446	0.198	−11.197 2.320
		Older × lauric acid	–7.409	–0.061	3.295	0.025	−13.873 −0.946
		Intercept	72.763		0.618	0.000	71.551 73.974
Myristic acid (g)			1.210	0.063	0.574	0.035	0.084 2.337
		Middle-aged	–12.784	–0.324	0.965	0.000	−14.676 −10.891
		Older	–24.892	–0.633	0.942	0.000	−26.740 −23.045
		Middle-aged × myristic acid	–1.122	–0.033	0.951	0.238	−2.987 0.743
		Older × myristic acid	–2.495	–0.076	0.903	0.006	−4.267 −0.723
		Intercept	72.714		0.627	0.000	71.484 73.944

Bootstrapping analyses revealed that the interaction terms for all nutrients were significant after 20,000 replications: older × energy (B 95% confident interval = −0.0051 −0.0003), older × maltose (−4.1208 −0.1482), middle-aged × lactose (−0.3508 −0.0283), older × lactose (−0.3685 −0.0707), older × calcium (−0.0099 −0.0007), older × butyric acid (15.0669 −2.6189), older × caproic acid (−23.3166 −3.5626), older × caprylic acid (−41.8170 −5.9221), older × capric acid (−18.3964 −2.7254) older × lauric acid (−13.8902 −0.9285) and older × myristic acid (−4.2723 −0.7185).

Table 9 shows the mean intake of nutrients that influence recollection performance that was significantly moderated by age group and the results from the one-way ANOVA conducted on these data. Nutrient consumption was higher in young adults than in middle-aged adults for all nutrients except maltose. Likewise, young adults’ intake was higher than that of older adults for energy, maltose, calcium, capric acid, lauric acid, and myristic acid. Additionally, middle-aged adults’ consumption of energy was higher than that for older adults. Reference daily intake and food sources for each of the nutrients that significantly influence episodic memory performance are listed in Table 10.

**TABLE 9:** Mean consumption of nutrients significantly moderated by age and one-way ANOVA results across groups.

	Young M(*SD*)	Middle-Aged M(*SD*)	Older M(*SD*)	*F* (2, 1547)	Tukey^a^ HSD
Energy (kcal)	2,584.58 (925.62)	2,348.95 (796.86)	2,173.62 (697.83)	33.44***	1≠2,3, 2≠3
Maltose (g)	1.76 (1.05)	1.66 (0.92)	1.58 (0.83)	4.84**	1≠3
Lactose (g)	14.80 (10.78)	12.47 (10.64)	13.67 (11.43)	5.83**	1≠2
Calcium (mg)	866.75 (364.30)	808.50 (356.87)	804.04 (378.00)	4.73**	1≠2,3
Butyric acid (g)	0.39 (0.26)	0.32 (0.25)	0.35 (0.27)	8.10***	1≠2
Caproic acid (g)	0.23 (0.16)	0.19 (0.16)	0.22 (0.17)	6.99***	1≠2
Caprylic acid (g)	0.13 (0.09)	0.11 (0.09)	0.12 (0.10)	8.15***	1≠2
Capric acid (g)	0.35 (0.21)	0.29 (0.21)	0.31 (0.22)	11.45***	1≠2,3
Lauric acid (g)	0.45 (0.27)	0.36 (0.25)	0.38 (0.26)	14.73***	1≠2,3
Myristic acid (g)	1.94 (0.95)	1.61 (0.95)	1.61 (0.97)	20.40***	1≠2,3

^a^ Significant difference among age groups (1 = young, 2 = middle-aged, and 3 = older) based on *post hoc* Tukey’s honest significance difference analyses.

**p < 0.01, ***p < 0.001.

**TABLE 10:** Reference daily intake and food sources of nutrients that influence recollection.

	Reference daily intake^a^	Food sources^b^
Protein (g)	50	Meat, fish, poultry, beans, nuts, dairy products, and soybeans
Fat (g)	78	Meat, chicken skin, dairy products, nuts, and cocoa butter
Cholesterol (g)	0.3	Pork, chicken, egg, fish, dairy products, and cold meats
Alcohol (g) women/men^c^	14/28	
Maltose (g)		Cereals, sweet potato, pears, broccoli, honey, and kiwis
Lactose (g)		Dairy products
Calcium (mg)	1,300	Dairy products, tofu, cornmeal, nuts, wheat, and salmon
Sodium (mg)	2,300	Cold cuts and cured meats, shrimp, chicken, and cheese
Heme (g)		Meat, poultry, and seafood
Alpha-tocopherol (mg)	15	Sunflower seed, almonds, hazelnuts, peanuts, and fish
Gamma-tocopherol (mg)		Soybean oil, walnuts, sesame seeds, pecans, and pistachios
Niacin (mg)	16	Cereals, salmon, tuna, meat, beef, peanuts, and chicken
Vitamin B6 (mg)	1.7	Garlic, rice flour, beans, sunflower seed, potatoes, and corn
Butyric acid (g)		Dairy products, some fermented food
Caproic acid (g)		Dairy products, vanilla, and gingko
Caprylic acid (g)		Dairy products, coconut oil, and palm kernel oil
Capric acid (g)		Dairy products, coconut oil, and palm kernel oil
Lauric acid (g)		Dairy products, coconut oil, and palm kernel oil
Myristic acid (g)		Nutmeg butter, coconut oil, palm kernel oil, beef, and salmon
Palmitic acid (g)		Palm oil, meat, dairy products, cocoa butter, and olive oil
Stearic acid (g)		Dairy products, red meat, margarine, and cocoa butter
Palmitoleic acid ω-7 (g)		Macadamia nut, sea buckthorn oil, and dairy products
Oleic acid ω-9 (g)		Olive oil, canola oil, meat, dairy products, and avocado
Gadoleic acid ω-11 (g)		Fish oil, mustard oil, beef tallow, corn, and arugula
Alpha-linoleic acid ω-3 (g)	1.6	Flaxseed, walnuts, chia seed, canola oil, and soybean
Linoleic acid ω-6 (g)	17	Sunflower oil, soybean oil, nuts, seeds, meat, and egg

*^a^United States Food & Drug Administration (2020).*

*^*b*^United States Department of Agriculture (USDA) (2018).*

*^*c*^Moderate alcohol intake, one drink for women and two for men [United States Department of Agriculture (USDA), 2015].*

## Discussion

A total of 19 nutrients significantly predicted recollection accuracy throughout the entire adult life span; these 19 nutrients included macronutrients, micronutrients and fatty acids, among others. However, neither carbohydrates nor carotenoids had any influence on episodic memory when the whole sample was considered. Remarkably, all significant nutrients had a positive effect on memory performance. The influence of only 10 nutrients on recollection performance was moderated by age; these nutrients included energy, carbohydrates, one macromineral and several saturated fatty acids. Notably, all these nutrients show a negative influence in older adults compared to that in young adults. Below, we discuss each of these findings in detail. The nutrients that showed effects on memory in the entire sample but that were moderated by age are discussed in the second section.

### Effects of Nutrients on Recollection Across the Adult Life Span

The total intake of protein and fat influenced recollection accuracy. The effect of proteins on memory could be explained by the fact that proteins consist of long chains of amino acids that are divided up during digestion, and some of these amino acids are precursors of neurotransmitters involved in memory (Bairy and Kumar, 2019). In particular, the amino acid glutamine is a precursor of glutamate, and the amino acids phenylalanine and thyrosine are precursors of dopamine, which in turn is a substrate of norepinephrine (Fernstrom, 1994). Fat is made up of fatty acids that break down during digestion, and fatty acids are involved in several processes within the brain, such as membrane fluidity, regulation of synaptic transmission and signal transduction (Chianese et al., 2018), functions that are important for all cognitive processes. Although cholesterol from the diet cannot pass through the blood brain barrier, several studies have examined the relationship between serum cholesterol levels and cognitive performance, but the results have been contradictory (for a review see Schreurs, 2010). The present data demonstrate that cholesterol from the diet benefits recollection; however, its effects may be indirect. One reason could be the function of cholesterol as a precursor of steroid hormones involved in memory, such as testosterone and estradiol (Pompili et al., 2020). A meta-analysis that included 143 studies concluded that moderate alcohol intake does not increase the risk of dementia or cognitive decline (Neafsey and Collins, 2011). The present findings further confirm that moderate alcohol intake has positive effects on episodic memory. The benefit of moderate alcohol intake is attributed to its anti-inflammatory properties that promote cellular survival mechanisms not only in the cardiovascular system but also in the brain (Collins et al., 2009).

Among micronutrients, the essential mineral sodium was positively associated with recollection accuracy. Sodium, the major cation electrolyte located in the extracellular fluid, is involved in cellular nerve transmission and in the maintenance of an adequate balance of fluids within the body (Strazzullo and Leclercq, 2014), functions that explain the influence of sodium not only on memory but also on all types of cognitive function. Heme, the kind of iron that comes from animal protein, is the component of hemoglobin responsible for binding oxygen that is transported by red blood cells from the lungs to the rest of the body. Additionally, as a component of cytochrome proteins, heme is involved in electron transport, which is necessary for adenosine triphosphate (ATP) synthesis. Therefore, heme deficiencies may reduce neuronal energy capacity, which may be manifested in memory or learning difficulties (Fretham et al., 2011). Here, we found that higher heme intake was associated with better episodic memory performance.

The consumption of the four tocopherol (α, β, γ, and δ) compounds of vitamin E positively influenced recollection accuracy. Although α- and γ-tocopherols are the predominant forms of vitamin E in plasma (Chow, 1975), all of the tocopherol types showed beneficial effects on memory. Tocopherols are potent antioxidants because they inhibit the production of free radicals that damage cell membranes. They act by donating hydrogen to free radicals, and in doing so they become oxidized; however, they are regenerated by other antioxidants (Traber and Atkinson, 2007). This outcome is in agreement with observational studies and trials that showed that vitamin E improved cognitive performance and reduced cognitive decline (for a review, see La Fata et al., 2014).

Two B vitamins were associated with better episodic memory performance. One of them was niacin (vitamin B3), which includes nicotinic acid and nicotinamide, precursors of coenzymes involved in redox reactions, cellular signaling, energy metabolism, DNA repair and apoptosis protection, among other functions (Gasperi et al., 2019). The effects of niacin on cognition have been predominately examined in neurodegenerative diseases. A longitudinal study that followed up 3,717 individuals for 5.5 years who were initially healthy showed that dietary niacin intake was associated with a lower incidence of cognitive decline and Alzheimer’s disease (Morris et al., 2004). Vitamin B6 (pyridoxine, pyridoxal, pyridoxamine and their phosphorylated forms) participates in the synthesis of several neurotransmitters, such as serotonin and dopamine, and in the metabolism of amino acids, fatty acids and homocysteine (Spinneker et al., 2007). Indeed, dietary vitamin B6 insufficiency or impaired metabolism of vitamin B6 due to inflammation increases plasma homocysteine levels, a condition associated with poor cognition and dementia (Seshadri et al., 2002).

Two saturated fatty acids (palmitic acid and stearic acid), three monounsaturated fatty acids (MUFAs) (palmitoleic acid ω-7, oleic acid ω-9, and gadoleic acid ω-11) and two polyunsaturated fatty acids (PUFAs) (alpha-linoleic ω-3, acid and linoleic acid ω-6) were positively associated with recollection accuracy. Although there is an extensive belief that saturated fatty acids are harmful because they increase total serum cholesterol, a meta-analysis of 60 trials revealed that lauric acid increases high-density lipoprotein, whereas myristic, palmitic and stearic acids had small or no effects on cholesterol lipid composition (Mensink et al., 2003). Nevertheless, moderate intake of saturated fatty acids has positive effects; for example, palmitic acid is involved in synaptogenesis and myelination processes (González and Visentin, 2016), and stearic acid protects hippocampal neurons in *in vitro* models of stroke (Wang et al., 2006).

The benefits of MUFAs were discovered in a study conducted in seven countries that led to the discovery that the Mediterranean diet was associated with lower blood cholesterol levels and, consequently, a lower incidence of cardiovascular diseases (Keys, 1980). Trials have confirmed that MUFA intake increases or maintains HDL and reduces low-density lipoproteins (Lopes et al., 2016); thus, MUFAs preclude cerebrovascular dysfunction. The importance of essential fatty acids ω-3 and ω-6 on cognition has been extensively investigated (Yehuda, 2012); the present findings confirm their benefits on episodic memory performance across the entire adult life span. PUFAs are constituents of cell membranes and participate in neural signaling as lipid secondary messengers that modulate several functions, such as neurotransmitter release (Haag, 2003).

### Nutrient Effects on Recollection Moderated by Aging

Most of the nutrients whose effects on recollection accuracy were moderated by aging had no significant effects in the entire sample, except for energy, lauric acid and myristic acid. In the case of energy, we found that the slope for the older adults differed from those of the other two age groups, and increasing kcal intake was associated with lower episodic memory accuracy only in older adults. Surprisingly, older adults consumed less energy than the other two groups. Therefore, the negative effects of energy intake on memory are not due to greater energy consumption in the older adults than in the other two aging groups. Instead, this outcome indicates that episodic memory would benefit from lower energy intake in older adults.

This finding is in agreement with the idea that a calorie-restricted diet increases longevity, protects the brain against oxidative stress and improves cognition (Hadem et al., 2019). The benefit of calorie restriction may be explained by the free radical theory that proposed that aging is the consequence of the damage caused by free radicals, unstable molecules that are the product of normal cellular metabolism (Harman, 1956). Thus, reducing the metabolic rate through dietary restriction reduces the production of free radicals. However, the reduction in neural oxidative stress is not the only benefit of calorie restriction. Recent evidence has shown that a low-calorie diet induces neurogenesis, reduces neuroinflammation and improves glucose regulation (Gillette-Guyonnet and Vellas, 2008; de Cabo and Mattson, 2019), which are mechanisms that may prevent cognitive deficits. Additionally, the rate of living theory may explain the benefit of a calorie-restricted diet, which is based on the observation that larger animal species live longer than small species, and this is attributed to slower metabolic rates in large species; in contrast, faster metabolic rates are associated with shortened life spans (Speakman, 2005). Thus, by reducing energy intake, the rate of energy metabolism would decrease, and age-related difficulties, such as cognitive decline, would be delayed.

Because fat provides more energy (9 kcal) than carbohydrates and protein, which provide only 4 kcal per gram (Tomassi and Merendino, 2006), the above interpretation may also apply to the finding that the influence of six saturated fatty acids (butyric acid, caproic acid, caprylic acid, capric acid, lauric acid and myristic acid) on episodic memory was moderated by age. The slopes in older adults for all these saturated fats differed from those in young adults. However, the slopes in middle-aged adults did not differ from those in the other two groups. In older adults, recollection accuracy decreased as the intake of these saturated fatty acids increased. In contrast, young adults’ recollection performance improved as the consumption of these saturated fatty acids increased. For middle-aged adults, the effect of the intake of these fatty acids on memory appeared to be neutral. In the present study, older adults’ consumption of butyric acid, caproic acid and caprylic acid did not differ from that of young adults, while middle-aged adults consumed less of all six fatty acids listed above than young adults. Therefore, saturated fatty acid intake in older adults was not reduced along with their expected biological changes, such as the decrease in energy regulation due to lower fat oxidation and energy expenditure, among others (Roberts and Rosenberg, 2006). The finding that excess saturated fatty acids have been linked to cognitive impairment and dementia (Barnard et al., 2014) may also explain the current results.

The negative effect of saturated fatty acid consumption on episodic memory in older adults may also be interpreted according to the gut-brain axis hypothesis. Several studies have identified that a high-fat diet influences the gut microbiota by decreasing *Bacteroidetes* and increasing *Firmicutes* (Oriach et al., 2016). These alterations induce energy harvest and storage, gut permeability and inflammation, which are conditions that lead to obesity. Due to strong gut-brain communication, obesity may lead to central nervous system disorders. Evidence supporting this association was provided by a study that showed that mice transplanted with microbiota from mice fed a high-fat diet demonstrated exploratory and cognitive impairments in the absence of obesity compared with control mice (Bruce-Keller et al., 2015).

The influence of two disaccharide carbohydrates, maltose and lactose, on memory was modulated by age. In both cases, the slope in older adults differed from that in young adults, but for lactose, the slope in middle-aged adults also differed from that in young adults. In older adults, the intake of both carbohydrates was associated with less recollection accuracy; in middle-aged adults, only the intake of lactose was negatively related to episodic memory performance. In contrast, young adults’ memory benefited from lactose intake. Maltose is composed of two glucose molecules, and after hydrolysis, the released glucose increases rapidly in human blood; indeed, due to hydrolysis, maltose has a glycemic index (105) higher than that of glucose (100) (Qi and Tester, 2020). For that reason, maltose could be involved in health issues, such as diabetes. In the current study, older adults consumed as much maltose as middle-aged adults; thus, it seems that the consumption was still higher considering the decreased energy regulation experienced in advanced age.

Lactose, composed of galactose and glucose, is only present in mammalian milk. The metabolite D-galactose, derived from lactose, has been extensively used in animal models to induce accelerated aging through oxidative stress (Azman and Zakaria, 2019). In a study that included 13,751 participants followed for 20 years, it was observed that participants who consumed more than one glass of milk per day showed a cognitive decline of 10% compared to individuals who hardly ever consumed milk (Petruski-Ivleva et al., 2017). In the present study, older adults consumed as much lactose as young adults, but the results revealed that lactose intake in these two groups has opposite effects on episodic memory. This difference might be explained by the fact that metabolism slows with age due to changes in body composition and hormone production, among other factors (Henry, 2000); these conditions may induce the accumulation of high levels of galactose, resulting in the production of reactive oxygen species. Remarkably, the negative effects of lactose intake on memory were observed beginning in middle age.

The effect of calcium on recollection accuracy was moderated by aging. The slope in older adults differed from that in young adults. Calcium intake in young adults was associated with better memory performance, but in older adults, the effects were inverted. This was found despite the fact that older adults consumed less calcium than young adults but not less calcium than middle-aged adults. Calcium is a critical signaling molecule that participates in numerous neuronal functions, such as neurotransmitter release, synaptic transmission, plasticity and memory (Kawamoto et al., 2012). The negative influence of calcium in the older group may be attributed to alterations in the regulation of calcium regulation. Normal brain aging results in changes in calcium homeostasis that alter several brain functions; for example, in hippocampal neurons, an increase in postsynaptic calcium impairs synaptic plasticity (Thibault et al., 2001). Although serum concentrations of calcium influence brain calcium levels (Harris et al., 1981), the cause of brain calcium dysregulation has been mainly attributed to other mechanisms, such as alterations in gene or protein expression (Kawamoto et al., 2012).

### General Remarks

One limitation of the present study was that nutrient intake was measured through the FFQ, an instrument that relies on participants’ ability to estimate the amount of food they consume. Although some inaccurate reports were anticipated, these errors are expected to be random across all food items. Another limitation is that we conducted a cross-sectional study that does not allow us to confirm conclusive causality among variables. However, because the FFQ examines dietary intake over a period of 1 year, we were able to reliably measure the influence of long-term dietary exposure on memory. This kind of information is not available through other precise methods that only provide short-term intake information.

The present study revealed which specific nutrients have a direct influence on recollection across the entire adult life span. Recollection is the most affected episodic memory process as individuals age, and its decline could be the prelude to more serious diseases. Although previous research has identified several nutrients that mainly influence general cognition (Gómez-Pinilla, 2008), the effects of nutrients have been analyzed individually and rarely together in the same sample as was done in the current study. This approach allowed us to provide a more complete overview of the nutrients that have the strength to impact brain functions involved in episodic memory.

The nutrients that are favorable for recollection across the adult life span, listed roughly according to the strength of their influence, are the PUFAs ω3 and ω6; vitamin E; stearic and palmitic saturated fatty acids; total fat intake; the oleic, palmitoleic and gadoleic MUFAs; alcohol; heme; the B vitamins niacin and B6; total protein; sodium; and cholesterol. Moreover, we identified which nutrient intakes should be reduced during specific life stages because their influence on memory is adverse. Additionally, ordered approximately according to the strength of their effects, recollection would benefit from lower consumption of lactose in middle-age and advanced ages; older adults would benefit from lower consumption of myristic, butyric, caproic, capric, caprylic and lauric saturated fatty acids; calcium; energy; and maltose.

## Data Availability Statement

The raw data supporting the conclusions of this article will be made available by the authors, without undue reservation.

## Ethics Statement

This study was approved by the Bioethics Committee of the School of Medicine at the National Autonomous University of Mexico. The patients/participants provided their informed consent to participate in this study.

## Author Contributions

SC wrote the manuscript, conceptualized, and designed the study. FT-T, CE-M, AF-M, and GR-P performed the experiments. CE-M organized the database. SC and SR-V analyzed the data. All authors contributed to the article and approved the submitted version.

## Conflict of Interest

The authors declare that the research was conducted in the absence of any commercial or financial relationships that could be construed as a potential conflict of interest.

## Publisher’s Note

All claims expressed in this article are solely those of the authors and do not necessarily represent those of their affiliated organizations, or those of the publisher, the editors and the reviewers. Any product that may be evaluated in this article, or claim that may be made by its manufacturer, is not guaranteed or endorsed by the publisher.
