# Supplementary material for: Influence of Dietary Nutrient Intake on Episodic Memory Across the Adult Life Span
Source: Front Aging Neurosci. 2021 Aug 30;13:724595. doi: 10.3389/fnagi.2021.724595 (PMC8435902; doi:10.3389/fnagi.2021.724595)
Supplement: Supplementary file 1 [file Table_1.pdf]

Supplementary Material

Table S1. Descriptive statistic results of all variables.

Variables	Mean	*SD*	Skewness	Kurtosis
Carbohydrates (g)	310.48	111.62	0.74	4.16
Protein (g)	82.16	29.89	1.00	4.88
Fats (g)	90.25	37.87	1.28	5.73
Energy (kcal)	2369.67	829.35	0.92	4.81
Cholesterol (g)[a]	5.46	0.56	-0.28	4.16
Alcohol (g)[a]	0.52	0.75	1.56	5.11
Caffeine (g)	141.51	171.34	1.80	6.38
Nitrate (g)[a]	4.19	0.57	0.02	3.51
Glucose (g)[a]	3.09	0.51	-0.12	3.32
Fructose (g)	31.45	17.09	1.84	9.04
Maltose (g)	1.66	0.94	1.58	6.69
Sucrose (g)	43.77	22.37	1.19	4.89
Lactose (g)	13.65	10.99	1.17	4.29
Starch (g)	86.62	44.23	1.32	8.53
Soluble fiber (g)	8.86	4.05	1.15	5.18
Insoluble fiber (g)	16.18	7.14	1.57	8.62
Calcium (mg)	826.59	367.40	0.83	3.87
Phosphorus (mg)	1332.03	478.60	0.66	3.60
Magnesium (mg)	360.35	123.17	0.72	4.23
Potassium (mg)	3517.30	1341.11	0.97	4.72
Sodium (mg)	1914.83	815.77	1.44	7.07
Copper (mg)[a]	1.25	0.41	0.80	4.44
Iron (mg)	13.75	5.13	1.18	5.86
Heme (g)[a]	0.63	0.25	0.60	4.51
Manganese (mg)[a]	2.69	0.85	-0.05	2.50

Selenium (mcg)	40.65	23.60	1.74	7.79
Zinc (mg)[a]	2.90	0.47	0.28	3.80
Retinol (UI)[a]	7.80	0.66	0.37	3.77
Vitamin D (UI)	207.33	127.97	1.16	4.67
Alpha-tocopherol (mg)[a]	2.55	0.40	0.26	3.66
Beta-tocopherol (mg)	0.74	0.40	1.55	7.21
Gamma-tocopherol (mg)	14.24	9.07	1.98	9.09
Delta-tocopherol (mg)[a]	1.16	0.42	0.57	3.64
Vitamin K (mcg)[a]	4.40	0.56	-0.02	3.46
Thiamine (mg)	1.74	0.71	1.20	5.80
Riboflavin (mg)	1.85	0.80	1.09	5.04
Niacin (mg)	20.76	7.81	1.20	5.70
Pantothenic acid (mg)[a]	2.44	0.58	0.43	3.38
Vitamin B6 (mg)*	1.83	0.77	0.22	2.80
Folate (mcg)[a]	6.58	0.74	0.18	3.05
Vitamin B12 (mcg)[a]	1.94	0.56	0.41	3.73
Vitamin C (mg)	232.76	142.71	1.73	8.46
Carotenes (UI)[a]	8.84	0.68	-0.42	3.33
Alpha-carotene (mcg)[a]	5.71	1.09	-0.68	3.59
Beta-carotene (mcg)	3691.17	2480.26	1.89	9.16
Lycopene (mcg)[a]	8.54	0.76	-0.71	6.32
Beta-cryptoxanthin (mcg)	687.61	595.43	1.87	8.35
Lutein & zeaxanthine (mcg)[a]	7.30	0.75	-0.22	3.53
Butyric acid (g)	0.35	0.26	1.09	4.31
Caproic acid (g)	0.21	0.17	0.95	3.95
Caprylic acid (g)	0.12	0.09	1.12	4.50
Capric acid (g)	0.32	0.22	1.06	4.34
Lauric acid (g)	0.40	0.26	1.18	5.54
Myristic acid (g)	1.72	0.97	0.93	4.04
Palmitic acid (g)	12.90	5.75	1.33	6.17
Stearic acid (g)	5.49	2.63	1.38	6.55

Palmitoleic acid ω-7 (g)	1.55	0.74	1.66	8.38
Oleic acid cis n-9 (g)	18.76	9.39	1.55	6.78
Gadoleic acid ω-11 (g)	0.16	0.09	1.85	8.56
Alpha-linoleic acid ω-3 (g)[a]	0.87	0.29	0.81	4.16
Eicosatetraenoic acid ω-3 (g)[a]	0.06	0.05	2.06	10.08
Eicosapentaenoic acid ω-3 (g)[a]	0.07	0.06	2.42	14.34
Docosahexaenoic acid ω-3(g)	0.18	0.16	1.98	8.25
Linoleic acid ω-6 (g)	9.23	5.13	1.79	7.79
Source memory (%)	60.32	18.54	-0.23	2.22

Note. [a] Log transformed variable
